# Blood Monocytes and Their Subsets: Established Features and Open Questions

**DOI:** 10.3389/fimmu.2015.00423

**Published:** 2015-08-17

**Authors:** Loems Ziegler-Heitbrock

**Affiliations:** ^1^Helmholtz Zentrum München, Asklepios Fachkliniken München-Gauting, Gauting, Germany

**Keywords:** monocyte subsets, nomenclature, classical monocytes, intermediate monocytes, non-classical monocytes

## Abstract

In contrast to the past reliance on morphology, the identification and enumeration of blood monocytes are nowadays done with monoclonal antibodies and flow cytometry and this allows for subdivision into classical, intermediate, and non-classical monocytes. Using specific cell surface markers, dendritic cells in blood can be segregated from these monocytes. While in the past, changes in monocyte numbers as determined in standard hematology counters have not had any relevant clinical impact, the subset analysis now has uncovered informative changes that may be used in management of disease.

## The Definition of Monocytes

The term monocyte is used for blood cells of a lineage called monocytes/macrophages or mononuclear phagocytes. These blood monocytes are bone marrow-derived leukocytes that are functionally characterized by the ability to phagocytose, to produce cytokines, and to present antigen. In early studies, they had been identified based on glass adherence and morphology (1). Also, cytochemistry for specific enzymes like monocyte-specific esterase (2, 3) has been employed, while the standard approach in clinical hematology relies on physical properties of these cells including light scatter.

In bone marrow, the monocytes derive from myelo-monocytic stem cells, which give rise to more direct precursors like monoblasts and pro-monocytes. These cells earlier were identified based on morphology (4) such that the monoblast was an ill-defined cell type. More recently in the mouse model, a Ly6C+ CD115+ CD117+ monoblast-type cell, termed common monocyte progenitor (cMoP), was identified in bone marrow and spleen and this cell is able to proliferate and give rise to the different monocyte subsets (5). A cMoP monoblast type of cell remains to be identified for man and other species.

The number of circulating blood monocytes in man can strongly increase within minutes by stress or exercise followed by a rapid return to baseline levels. These recruited cells are thought to come from what is called the marginal pool (6). This compartment describes areas of reduced blood velocity close to the endothelium of venules and here cells can loosely adhere and can be mobilized in a catecholamine-dependent fashion (7). These marginal pool monocytes can have an adhesion molecule pattern distinct from monocytes found in blood at rest.

In addition, CD11b^high^ (CD90, B220, CD49b, NK1.1, Ly-6G, F4/80, I-Ab, CD11c)^low^ cells are mobilized from the spleen after severe injury (8). These cells have monocyte morphology and their transcriptome matches with that of blood monocytes. Furthermore, CD11b+ Ly6C^hi^ monocytes can be mobilized from bone marrow to blood in infectious disease models (9), and adoptively transferred monocytes were shown to return to the bone marrow (10) in the mouse. What remains to be determined is whether the spleen and bone marrow compartments also contribute to the pool of monocytes that can be mobilized by stress and exercise.

When under homeostatic or inflammatory conditions, the monocytes have migrated into tissue; then by definition, these cells are called macrophages. Cells newly emigrated into the lung have been termed monocytes in some studies [e.g., Ref. (11)]. Since monocytes, once they have arrived in tissue, will start to transform into larger cells and rapidly lose their monocyte characteristics, others have called these recently emigrated cells “small macrophages” (12).

More detailed studies in the mouse have demonstrated tissue cells with characteristics close to blood monocytes (13, 14). However, these cells in the lymph node show a gene expression pattern that distinguishes them from the blood cells (14) and in the skin they show increased expression of lysozyme and CD68, markers typical of mature macrophages (13). Therefore, more data are required in the mouse model and obviously also in man before a consensus can be reached whether we use the term tissue monocyte or whether we continue to call these cells macrophages. Until these issues have been resolved, the term monocyte should be restricted to cells in the blood compartment and the bone marrow and spleen reservoirs that can replenish the blood monocyte pool.

## Definition of Blood Monocytes Based on Cells Surface Markers

As explained above, monocytes initially had been identified by function and morphology and these criteria have been misleading especially when disease processes altered these features. Therefore, attempts have been made to define unequivocal criteria for monocytes. Here, monoclonal antibodies against cell surface molecules have been proposed. In man, CD14 has been used as a marker (15), and in the mouse, CD115 is often employed (16). CD115 identifies the M-CSF receptor and has the main drawback that in the mouse, it is downregulated on blood monocytes with inflammation (17). Also, the question is whether such markers are sufficiently specific and do not react with other cell types like dendritic cells (DCs). In fact, part of the CD1c+ blood DCs in man can express low-level CD14 (18) and also human B cells have been reported to express some CD14 (19). Therefore, monocytes can be identified with markers like CD14 and CD115, but this should be supported by additional markers and by functional studies. Interestingly, when searching for macrophage-specific transcripts in the mouse, CD64 and MerTK have emerged (20). While CD64 is absent from non-classical monocytes in man, MerTK is a molecule that might prove informative for blood monocytes in different species. In addition, staining for CD16, which is used for monocyte subset definition (see below), will at the same time help to exclude DCs in human blood.

## Dissection of Monocytes from Dendritic Cells

Dendritic cells were first described by Steinman and Cohn as stellate cells isolated from mouse spleen (21). Over the years, there have been debates as to whether these cells are a distinct lineage or part of the mononuclear phagocyte system. A common precursor for monocytes and DCs was described in the mouse (22), but the existence of this cell was later disputed (23) suggesting that DCs and monocytes may diverge at an earlier multi-potent progenitor stage (24).

However, the demonstration that monocytes can be used to generate DCs *in vitro* by adding GM-CSF and IL-4 suggested a close relationship between monocytes and DCs (25). Later, transcriptome analysis demonstrated that such monocyte-derived DCs rather resemble macrophages than DCs from lymphoid tissue (26). Therefore, these *in vitro* generated monocyte-derived cells are potent antigen-presenting cells, but they do not represent *bona fide* DCs; they rather belong to the monocyte/macrophage lineage. Still not resolved is the question whether in tissue the monocyte-derived cells with high levels of class II expression and with high antigen-presenting capacity should be termed monocyte-derived DC (13, 27, 28) or activated macrophages.

In addition to DCs in tissue, cells with DC properties have been described in blood based on the expression of CD68, CD1c, or CD141 (29, 30). Transcriptome analysis has demonstrated that these cells and the monocytes belong to different clusters (26, 31). These data suggest that blood DCs can be segregated from monocytes and macrophages as a separate lineage.

The data also demonstrate the power of transcriptomic analysis in defining and dissecting leukocyte populations like monocytes and DCs. Ontogeny can help in such a definition, but in men, adoptive transfer is limited to strategies like transfer of bone marrow stem cells, and informative mutations are rare. Also, the ontogeny approach needs to be used with caution since a defined progenitor cell can give rise to clearly distinct cell populations. An informative example is the megakaryocyte–erythrocyte progenitor (MEP) cell, which gives rise to either megakaryocytes and their platelet progeny or to erythroblasts and their red blood cell progeny (32, 33). Megakaryocytes and erythroblasts have a distinct transcriptome (34), and they are involved in distinct functions, i.e., in blood clotting and oxygen transport, respectively. Therefore, although having a common ontogeny, these cells belong to clearly separate lineages. This example illustrates that ontogeny can provide a framework, but a comprehensive analyses like transcriptomics and the analysis of cell function are required for dissecting cell types and for developing a nomenclature. Therefore, in order to assign a novel leukocyte population in blood or tissue to either monocytes or DCs, a straight-forward approach is to analyze the transcriptome (and other omics like the proteome, lipidome, glycome, or metabolome) of these cells in comparison to typical monocytes and DCs and to then ask whether the novel cell type co-clusters with either prototypic monocytes or DCs (26).

## Monocyte Subpopulations

Evidence for monocyte subpopulations has come from experiments using differential flotation in counter-current elutriation (35) and from differential binding to antibody-coated red blood cells, which has defined populations with different functions (36). With the use of monoclonal antibodies and flow cytometry, tools have become available to clearly define, enumerate, and isolate monocyte subsets based on the differential expression of CD14 and CD16 cell-surface markers (37).

**Figure 1:**
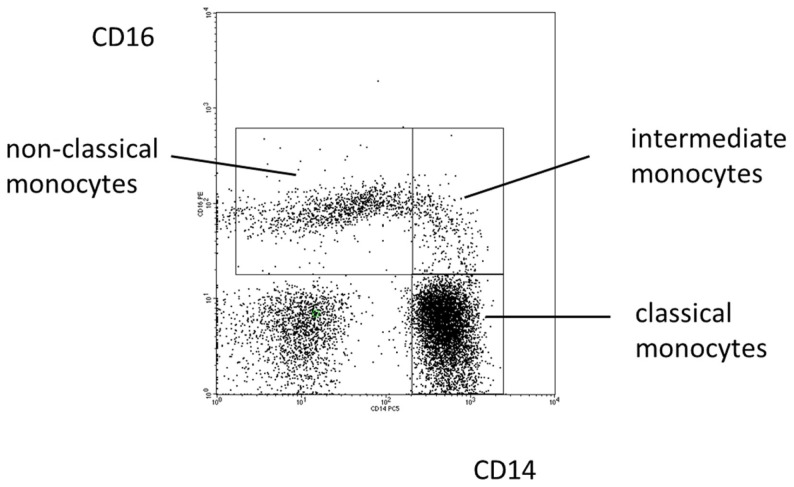
**Blood monocyte subsets in man**. Illustration of the definition of human monocyte subsets in health based on a typical distribution of events in a CD14 CD16 staining.

In 2010, an international consortium under the auspices of the IUIS and the WHO has proposed a nomenclature for monocyte subpopulations (38). The proposal defined the major population of CD14^high^ cells found in human blood as classical monocytes and the minor population of cells with low CD14 and high CD16 as non-classical monocytes. A population in between these two subsets was termed intermediate monocytes (see Figure 1).

While an unequivocal approach to defining the intermediate monocytes has not been developed, as yet (39), a host of studies on intermediate monocytes has been published since the 2010 proposal. In fact, a search for the term “intermediate monocyte” under Google Scholar has revealed more than 100 studies on these cells since 2010. These reports have described an expansion of intermediate monocytes in various inflammatory diseases and these cells have been shown to be of prognostic relevance in cardiovascular disease (40). The use of additional markers for delineation of intermediate monocytes has been suggested (41) and it remains to be shown whether markers, such as CCR2 or slan, will improve the definition of these cells.

The same nomenclature as proposed for man can be used in other species [reviewed in Ref. (42)]. The respective cells can be very similar to men as seen for non-human primates (43, 44). In species like the mouse, the classical and non-classical monocyte subsets can be identified as well, but different markers like CD115, Ly6C, and CD43 are used (16, 45). Also in species like rat, pig, cow, and horse, classical and non-classical monocytes can be defined and even intermediate monocytes have been described in some animals (42). It is predicted that the nomenclature of monocyte subsets will be applicable to all mammalian species.

In human blood, a population of slan-positive cells has been described as DCs, but phenotypic analysis has shown that these cells are CD14-low and CD16-high (46), functional studies demonstrated a high capacity to produce TNF (47), and clinical studies showed that these cells are depleted by glucocorticoid treatment (48). These features are identical to what has been reported as characteristics of non-classical CD14+CD16++ monocytes (37, 49, 50). Also, the increased absolute numbers of slan-positive monocytes and of non-classical monocytes show a clear correlation in HIV-infected patients (51), and part of the non-classical monocytes has been shown to be slan-positive (52–54). Collectively, these findings suggest that the slan-positive cells belong to the non-classical monocytes.

There may be additional monocyte subsets including Fcepsilon-RI-positive cells (55), which were found with a median of 2.5% among CD14-positive blood monocytes in a pediatric cohort (56) and these cells may be involved in IgE clearance (57). Also, proliferating monocytes have been described (58) as well as precursors for fibrocytes (59) and osteoclasts (60). For all of these cell types, further characterization is awaited.

## Clinical Implications of Monocyte Numbers

Monocyte numbers as defined in the hematology lab using light scatter properties have not contributed much to diagnosis and monitoring of disease, but with the definition of monocyte subsets by flow cytometry, informative patterns have emerged. For example, severe infection will increase the number of non-classical and intermediate monocytes (61–63). Here, it remains to be analyzed whether such an increase can predict prognosis, as has been suggested (64). Furthermore, therapy with glucocorticoids leads to a decrease of non-classical monocytes, which appears to be due to a selective induction of apoptosis in the non-classical monocytes while classical monocytes even increase in number under glucocorticoids (50, 65). Also, blockade of the M-CSF pathway can lead to depletion of non-classical monocytes (66–68). A likely explanation is that M-CSF signaling via the CD115 M-CSF receptor is required for the classical monocytes to mature into non-classical monocytes. Again still to be determined is whether such a drug-induced depletion can be used to predict therapeutic response in inflammatory diseases. Still unresolved is the mechanism of depletion of non-classical monocytes in three siblings within one family (69). Here, more families with this type of defect need to be analyzed in order to identify the gene and the mechanisms involved. Finally, the absolute count of intermediate monocytes was shown to predict cardiovascular events (70, 71). Hence, analysis of monocyte subsets by flow cytometry now provides clinically useful parameters in various settings. What remains to be established in this context is an unequivocal dissection of the non-classical and the intermediate monocytes.

## Conflict of Interest Statement

The author declares that the research was conducted in the absence of any commercial or financial relationships that could be construed as a potential conflict of interest.
